# Genesis of the Master Circadian Pacemaker in Mice

**DOI:** 10.3389/fnins.2021.659974

**Published:** 2021-03-23

**Authors:** Arthur H. Cheng, Hai-Ying Mary Cheng

**Affiliations:** ^1^Department of Biology, University of Toronto Mississauga, Mississauga, ON, Canada; ^2^Department of Cell and Systems Biology, University of Toronto, Toronto, ON, Canada

**Keywords:** suprachiasmatic nucleus, neurogenesis, development, neuronal differentiation, transcription factors, neuropeptides

## Abstract

The suprachiasmatic nucleus (SCN) of the hypothalamus is the central circadian clock of mammals. It is responsible for communicating temporal information to peripheral oscillators via humoral and endocrine signaling, ultimately controlling overt rhythms such as sleep-wake cycles, body temperature, and locomotor activity. Given the heterogeneity and complexity of the SCN, its genesis is tightly regulated by countless intrinsic and extrinsic factors. Here, we provide a brief overview of the development of the SCN, with special emphasis on the murine system.

## Introduction

On this rhythmic planet, we are surrounded by countless environmental oscillations of varying frequencies. The most notable of all rhythms is the 24 h day-night cycle, which results in predictable changes in the availability of light, warmth, and sustenance. In order to thrive, organisms must be able to anticipate and prepare for daily rhythmic events in the environment, rather than simply reacting to them upon their detection. From bacteria to humans, organisms evolved circadian clocks to keep track of time, enabling them to prepare for external challenges and opportunities through temporal coordination of behavior and physiology.

In mammals, circadian rhythms are driven by a hierarchy of tissue-specific oscillators throughout the body, orchestrated by a central circadian pacemaker, the hypothalamic suprachiasmatic nuclei (SCN). Individual SCN neurons synthesize neuropeptides and neurotransmitters to coordinate endogenous oscillations and entrainment at the tissue level (Aton et al., 2005; Maywood et al., 2011; Mieda et al., 2015). The SCN is unique within the clock hierarchy, as it is the only clock to respond to light directly (Morin and Allen, 2006). The SCN (oscillator) receives and integrates time cues (input) from the environment, and communicates temporal information to peripheral oscillators via humoral and endocrine signaling. Ultimately, the SCN coordinates and controls overt rhythms (outputs) such as sleep-wake cycles, body temperature, osmoregulation, hormone secretion, and gastrointestinal, hepatic, and cardiac functions (Roenneberg and Merrow, 2016). As the biological clock coordinates nearly all physiological processes, perturbation of the circadian system constitutes a risk factor for a myriad of disorders, including obesity, diabetes, cardiovascular disease, cancer, and neurodegeneration (Kurose et al., 2011; Uth and Sleigh, 2014; Broussard and Van Cauter, 2016; Melo et al., 2016; Khaper et al., 2018; Musiek et al., 2018). Conversely, many pathological conditions (e.g., Alzheimer’s disease, cancer) contribute to circadian disruption, which further exacerbates them (Lim et al., 2014). By expanding our knowledge and mechanistic understanding of the circadian clock, we position ourselves to develop new strategies that leverage the circadian system to promote better physical and mental health.

## Transcription-Translation Feedback Loop/The Molecular Clock

**FIGURE 1:**
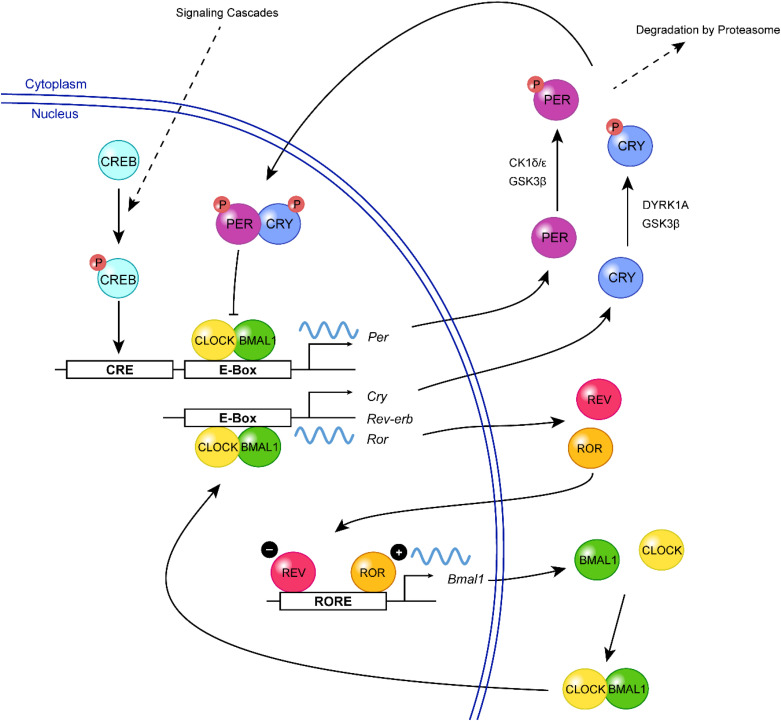
A simplified view of the mammalian molecular clock. In the positive limb of the primary feedback loop, CLOCK (yellow) and BMAL1 (green) form a heterodimer and bind to the E-box elements in the promoter regions of *Per* and *Cry*, triggering their transcription. Following their translation, PER (purple) and CRY (blue) proteins are phosphorylated (red, P) by various kinases including CK1δ/ε, GSK3β, and DYRK1A, which can regulate their turnover or nuclear entry. In the negative limb, PER:CRY heterodimers translocate to the nucleus, where they inhibit CLOCK:BMAL1-mediated transcription, thereby repressing their own gene expression. The transcription of *Bmal1* is further regulated by a second feedback loop involving two E-box-regulated genes, *Rev-Erb* and *Ror*. REV-ERB (magenta) inhibits the transcription of *Bmal1* by competing with the transcriptional activator, ROR (orange), for binding of the ROR-element within the *Bmal1* promoter. Extracellular signals (e.g., neurotransmitters, neuropeptides) can activate signaling cascades resulting in the phosphorylation of CREB (turquoise), which mediates *Per* transcription and resetting of the clock.

The molecular clock machinery relies on a series of transcription-translation feedback loops (TTFLs) that generate rhythmic expression of “clock genes” through a negative feedback mechanism. In mammals, the positive limb of the core feedback loop consists of the basic helix-loop-helix PAS transcription factors, Circadian locomotor output cycles kaput (CLOCK) and Brain and muscle ARNT-like protein 1 (BMAL1) (Figure 1). During the subjective day, CLOCK and BMAL1 dimerize and bind to the E-box elements in Period (*Per1*, *Per2*) and Cryptochrome (*Cry1*, *Cry2*) promoters, inducing their transcription (Shearman et al., 1997; Gekakis et al., 1998; Kume et al., 1999; Bunger et al., 2000; Vitaterna et al., 2006). During the subjective night, PER and CRY protein heterodimers translocate from the cytoplasm to the nucleus, where they repress their own gene transcription through inhibition of CLOCK:BMAL1 (Kume et al., 1999; Vitaterna et al., 1999; Zheng et al., 1999, 2001; Shearman et al., 2000). PER proteins are degraded during the late subjective night, but CRY continues to accumulate in the nucleus and maintains the repressive phase of the cycle (Ye et al., 2014). In the early subjective day, the degradation of CRY results in the derepression of CLOCK:BMAL1-mediated transcription and thus a new round of E-box-dependent gene expression (Gekakis et al., 1998; Kume et al., 1999). This core clock circuitry is regulated by secondary feedback loops. For example, the transcription of *Bmal1* is positively and negatively regulated by the nuclear orphan receptors, ROR (α, β, and γ) and REV-ERB (α and β), respectively, which are themselves E-box-containing genes and are therefore controlled by the primary feedback loop (Preitner et al., 2002; Sato et al., 2004; Guillaumond et al., 2005). Together, the primary and secondary TTFLs drive the ∼24 h oscillation of the molecular clock, while additional layers of regulation (described in the following sections) ensure its stability and robustness.

## SCN Structure and Connectivity

As it is currently understood, the SCN is responsible for interpreting photic and non-photic signals that it receives from afferent projections, and ultimately produces a coherent temporal output to peripheral oscillators through humoral and neuroendocrine mechanisms. Each individual SCN neuron harbors the clock machinery and is able to maintain robust molecular rhythms on a single-cell level. Through neuropeptide, neurotransmitter, and synaptic signaling, SCN neurons form an intricately connected oscillatory network with astounding precision and resilience.

**FIGURE 2:**
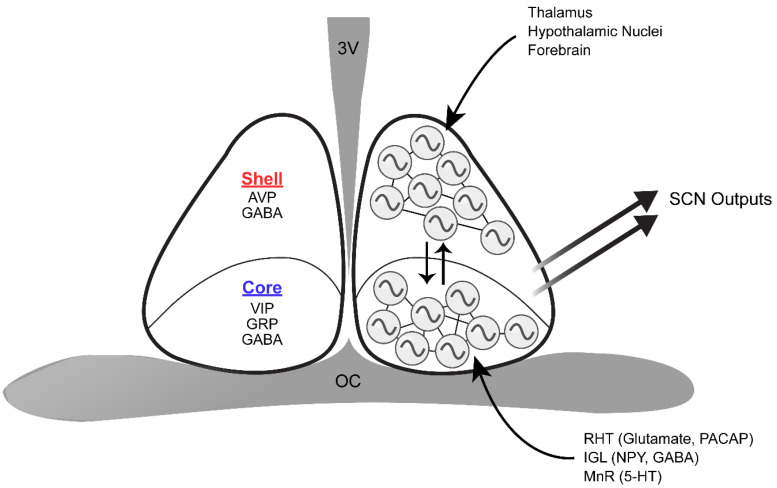
Schematic of the structure and organization of the SCN. The dorsomedial SCN (shell) expresses AVP and GABA, whereas the ventrolateral SCN (core) synthesizes VIP, GRP, and GABA. The retinohypothalamic tract (RHT), intergeniculate leaflet (IGL), and median raphe nucleus (MnR) directly innervate the core. On the other hand, inputs from the thalamus, various hypothalamic nuclei, and the forebrain are mainly received in the shell. Core and shell SCN neurons are synchronized through various means of intercellular communication, and are thus capable of producing coherent outputs to peripheral clocks. 3V, third ventricle; OC, optic chiasm.

The SCN is a pair of nuclei located in the anterior hypothalamus, situated directly dorsal to the optic chiasm and lateral to the third ventricle. It is comprised of approximately 20,000 heterogenous neurons that secrete dozens of neuropeptides, neurotransmitters, and cytokines, many of which can be at least partially co-expressed by certain populations of SCN neurons (Figure 2; Abrahamson and Moore, 2001; Cheng et al., 2002; Antle and Silver, 2005; Todd et al., 2020; Wen et al., 2020). The SCN is classically divided into two subregions, a light-responsive ventrolateral “core” and a rhythmic dorsomedial “shell,” based on the neurochemical nature of cells in each area and its physiological function (Aton et al., 2005). SCN core neurons are characterized by expression of vasoactive intestinal peptide (VIP), gastrin releasing peptide (GRP), calbindin, calretinin, neuromedin S (NMS), and neurotensin (Abrahamson and Moore, 2001; Lee et al., 2015). In contrast, SCN shell neurons express arginine vasopressin (AVP), calbindin, NMS, angiotensin II, and met-enkephalin (Abrahamson and Moore, 2001; Lee et al., 2015). All SCN neurons synthesize γ-aminobutyric acid (GABA) as the main neurotransmitter in addition to the neuropeptidergic signals (Moore and Speh, 1993; Abrahamson and Moore, 2001).

In addition to neurons, astrocytes in the murine SCN also contribute to circadian timekeeping. Astrocytes have been shown to display daily rhythms in structural protein expression, morphology, metabolic function, and clock gene expression (Prolo et al., 2005; Becquet et al., 2008; Cheng et al., 2009; Burkeen et al., 2011). Astrocyte-specific ablation of *Bmal1* lengthens the period of clock gene oscillations and locomotor behavior (Barca-Mayo et al., 2017; Tso et al., 2017). Furthermore, excision of the short-period CK1ε tau mutation specifically from SCN astrocytes lengthens molecular and behavioral rhythms (Brancaccio et al., 2017; Tso et al., 2017). It has been shown that SCN astrocytes control circadian period by regulating GABA uptake and glutamatergic signaling (Barca-Mayo et al., 2017; Brancaccio et al., 2017, 2019). Recently, Sominsky et al. (2021) reported that microglia are another important component for maintaining clock gene expression and behavioral rhythms. By expressing the diphtheria toxin (DT) receptor specifically in fractalkine receptor-positive cells (*Cx3cr1*^+^), 94% of SCN microglia was acutely ablated in transgenic Wistar rats (Sominsky et al., 2021). This resulted in pronounced disruption of behavioral rhythms, circadian temperature profiles, and *Per1* and BMAL1 expression (Sominsky et al., 2021).

As the master circadian clock, the SCN is intricately connected with many regions of the brain to regulate the phase and period of circadian rhythms. The SCN has three major afferent connections: retinohypothalamic tract (RHT) projections from the retina, geniculohypothalamic tract (GHT) projections from the intergeniculate leaflet (IGL), and serotonergic projections from the median raphe nucleus (MnR) in the brainstem (Meyer-Bernstein and Morin, 1996; Mintz et al., 1997; Abrahamson and Moore, 2001). Photic information received by intrinsically photosensitive retinal ganglion cells (ipRGCs) is delivered to the SCN through the RHT (Do and Yau, 2010). Although the terminal fields of the RHT can be found in all parts of the murine SCN, the core region has denser retinal innervation compare to the SCN shell (Morin et al., 2006). IpRGC input is essential for SCN light entrainment, which occurs through the release of glutamate, aspartate, and the neuropeptide pituitary adenylate cyclase-activating polypeptide (PACAP) (Chen et al., 1999; Guido et al., 1999; Kawaguchi et al., 2003; Hannibal et al., 2008). In contrast, the IGL innervates the SCN with neuropeptide Y (NPY) and GABA terminals (Moore et al., 2002). As with RHT terminals, NPY terminals are concentrated in the ventro-central region of the SCN and are sparser in the dorsomedial region (Morin et al., 2006). The IGL plays a significant role in relaying photic and non-photic information to the SCN, as either IGL lesions or NPY infusion into the SCN can alter circadian rhythms (Albers and Ferris, 1984; Pickard et al., 1987). The SCN also receives serotonergic projections from the MnR, where the plexus appears to be densest along the medial and ventral SCN border, grading to sparse innervation centrally and dorsolaterally (Morin et al., 2006). Notably, all three major SCN inputs significantly overlap in the core SCN, reflecting its key role in integrating luminance information from the retina and non-photic input from the midbrain arousal center during entrainment. In addition to these three major afferents, ∼35 brain regions have been shown to project to the SCN: these include other hypothalamic nuclei, the amygdalohippocampal zone, and brainstem nuclei (Krout et al., 2002). However, relatively few SCN afferent systems have been explored in terms of their rhythm-related functions. On the other hand, SCN efferents innervate ∼15 brain regions, likely carrying circadian rhythm phase information to distal targets. Notably, the SCN projects to many hypothalamic nuclei including the preoptic area, the paraventricular nucleus, the subparaventricular zone, the retrochiasmatic area, the dorsomedial and ventromedial nuclei, and the premammillary area (Watts and Swanson, 1987; Abrahamson and Moore, 2001). These efferent projections from the SCN have been implicated in circadian regulation of body temperature, locomotor activity, sleep-wake cycles, and feeding (Lu et al., 2001; Chou et al., 2003; Abrahamson and Moore, 2006). Neuropeptides such as prokineticin 2 (PROK2) have been shown to serve as functional outputs of the SCN, communicating phase information to other brain regions (Cheng et al., 2002). Mice deficient in either PROK2 or its cognate receptor, prokineticin receptor 2 (PROKR2), have a pronounced redistribution of locomotor activity from early night to late night with significantly dampened amplitude (Li et al., 2006; Prosser et al., 2007).

## Intra-SCN Communication and Signaling

*In vivo* or in organotypic cultures, oscillations of SCN neurons are synchronized and coherent, yet follow a consistent pattern of distinct phases and amplitudes. Bioluminescence imaging of SCN explants *in vitro* using *Per1*-LUC or PER2:LUC reporters have shown that the shell region has much more pronounced PER oscillations than the core (Yamaguchi et al., 2003). Circadian cycling of PER expression begins in the dorsomedial periventricular region of the shell, propagates ventrally and laterally to the center of the shell after 4–8 h, and ends in the ventral SCN after 12–15 h (Yamaguchi et al., 2003). However, a minority of cells have been shown to remain ∼12 h out-of-phase with the population mean, potentially allowing multiple, variously phased output signals to be generated (Herzog et al., 1997; Nakamura et al., 2001). This oscillation pattern is preserved from cycle-to-cycle, as well as after pharmacological manipulations that delay or stop the clock, suggesting that SCN coupling is mediated by specific neural circuits instead of a homogeneous coupling scheme (Liu et al., 1997). When challenged by an abrupt phase shift (jet lag), the shell and core SCN exhibit desynchronization and appear to be out-of-phase initially (Nagano et al., 2003; Albus et al., 2005; Nakamura et al., 2005). The phase of the ventral region, measured by either clock gene expression or impulse activity of SCN neurons, shifts rapidly, whereas the dorsal region requires more days to shift and to align with the ventral SCN (Nagano et al., 2003). This finding is consistent with the notion that the core SCN is the compartment that receives photic cues and directs resynchronization of the shell. When cultured at low density, individual SCN neurons can still express robust rhythms autonomously for weeks, showing that they do not require rhythmic input from other cells to oscillate (Welsh et al., 1995). However, dispersed clock cells in the same culture dish display rhythms with varying periods and progressively distinct phase relationships. Dissociating SCN cells thus removes the coupling forces that normally maintain intercellular synchrony at the tissue level.

Three modes of intercellular communication have been established to maintain network stability and coupling of the SCN: chemical synapses, electrical synapses (gap junctions), and paracrine signaling. The most common neurotransmitter in the SCN is GABA, which is present in all SCN neurons (Moore and Speh, 1993). Although GABA usually elicits spontaneous inhibitory post-synaptic potentials, it may be excitatory in some instances (De Jeu and Pennartz, 2002; Albus et al., 2005; Hee et al., 2008). GABA-mediated excitation is most common at night in the dorsal region of the SCN, a process mediated by Na^+^-K^+^-Cl^–^ cotransporter 1 (NKCC1) and K^+^-Cl^–^ cotransporters (KCCs) (De Jeu and Pennartz, 2002; Hee et al., 2008). In Syrian hamsters, blocking excitatory responses of GABA by inhibiting NKCC1 has been shown to attenuate light-induced phase delays during the early subjective night (McNeill et al., 2018). In the SCN, many cellular responses of GABA are driven by ionotropic GABA_A_ and Gα_i/o_−coupled GABA_B_ receptors (Jiang et al., 1997b; Strecker et al., 1997; Liu and Reppert, 2000; Gribkoff et al., 2003; Belenky et al., 2008). GABA_A_ receptor-mediated signaling causes phase shifts when applied to dissociated SCN neurons, and daily pulses of GABA synchronizes them (Liu and Reppert, 2000). In addition, GABA signaling is essential for shell and core re-synchronization after a jet lag treatment, as well as for modulating phase shifting responses after a light pulse (Ralph and Menaker, 1985, 1986, 1989; Gillespie et al., 1997, 1999; Albus et al., 2005; Albers et al., 2017). On the other hand, blocking both GABA_A_ and GABA_B_ receptors does not affect oscillatory amplitude and synchrony of neurons in SCN slices (Aton et al., 2006). Recently, Barca-Mayo et al. (2017) showed that astrocyte-mediated GABA signaling modulates clock gene expression of cortical neurons *in vitro* (Barca-Mayo et al., 2017).

It is widely accepted that communication between neurons is mediated primarily by Ca^2+^-dependent synaptic transmission. However, when chemical synaptic transmission is blocked using Ca^2+^-free medium, periodic and synchronized bursts of action potentials in a large population of SCN neurons can still be detected, indicating that mechanisms other than chemical synaptic transmission may modulate SCN synchrony (Bouskila and Dudek, 1993). It was also noted that metabolic rhythms in the embryonic SCN precede chemical synaptogenesis in rats, indicating that non-synaptic mechanisms may be important in coordinating circadian rhythms (Reppert and Schwartz, 1984). Subsequently, gap junctions (electrical synapses) have been identified in the SCN and found to mediate neuronal coupling (Jiang et al., 1997a; Colwell, 2000; Long et al., 2005; Rash et al., 2007; Wang et al., 2014). Gap junction channels allow the passage of ions and other small molecules between coupled cells and function to connect cells electrically and metabolically. They are formed by two hemichannels, each composed of 6 connexin proteins (Cheung et al., 2014). The majority of neuronal gap junctions in the SCN are miniature gap junctions that are composed of less than 50 connexons comprised primarily of connexin-36 (Cx36) subunits (Rash et al., 2007). Possibly due to the small number of large gap junctions and the predominance of mini-gap junctions, there is limited electrotonic coupling and coupling-mediated spike-for-spike synchronization between SCN neurons (Rash et al., 2007). Cx36 knockout mice have deficits in circadian behavior and electrical coupling between SCN neurons; however, the results are complicated by the global nature of the Cx36 ablation (Long et al., 2005). Blocking gap junctions in SCN slices with carbonoxolone also weakens synchrony of the SCN network (Wang et al., 2014).

The SCN expresses a plethora of neuropeptides, many of which are strongly implicated in SCN coupling. The core SCN and the most prevalent neuropeptide intrinsic to this region—VIP—are vital for maintaining coupling within the SCN. In mice, VIP is released rhythmically from the core and acts through the G-protein coupled, vasoactive intestinal peptide receptor 2 (VPAC2, also known as VIPR2), which is expressed in both the core and the shell SCN (Shinohara et al., 2000; Dardente et al., 2004). *Vip* or *Vpac2* knockout mice display weak behavioral rhythms and often become arrhythmic after a few days in constant darkness (Harmar et al., 2002; Colwell et al., 2003; Aton et al., 2005). Organotypic SCN slices from these mice show suppressed neuronal firing, low amplitude clock gene rhythms, and desynchrony among cells (Cutler et al., 2003; Aton et al., 2005; Brown et al., 2007; Hughes et al., 2008; Maywood et al., 2011). VIP evokes phase shifts in locomotor activity, AVP release, multiunit firing rate, and PER2:LUC rhythms in a phase- and dose-dependent manner (Piggins et al., 1995; Watanabe et al., 2000; Reed et al., 2001; An et al., 2011). Molecularly, VIP-mediated phase shifting requires PKA, PLC, and MAPK signaling pathways, which ultimately converge on the activation of CRE-mediated transcription of clock genes (Nielsen et al., 2002; Meyer-Spasche and Piggins, 2004). Furthermore, VIP has been shown to modulate the strength of electrical synapses, which in turn regulate intercellular coupling (Wang et al., 2014). Intriguingly, studies have demonstrated that VIP can promote network plasticity by destabilizing intercellular synchrony in addition to its role as a synchronizing factor. For instance, application of exogenous VIP at concentrations greater than 100 nM desynchronizes and broadens the phase distribution of cells within the SCN, in particular during the nadir of PER2 expression (An et al., 2013). When microinjected into the SCN during the early subjective day, a phase when VIP does not induce phase shifts, VIP accelerates entrainment of locomotor rhythms to an advanced LD cycle in a jet lag paradigm (An et al., 2013). Although *Vip^–/–^* and *Vpac2^–/–^* mice have weak behavioral rhythms in DD, their behavioral rhythmicity can be restored by long-term exposure to constant light (An et al., 2013; Hughes et al., 2015). Detailed analyses of *Vpac2^–/–^* mice revealed that exposure to LL diminishes the intercellular signaling deficit in these animals, resulting in both improved behavioral rhythms and increased cellular synchrony (Hughes et al., 2015). This is in stark contrast with the disruptive effect of LL on neuronal function and physiological rhythms in animals with a fully functional SCN, where the elevated level of VIP induced by LL might destabilize the circadian pacemaker (Ohta et al., 2005; An et al., 2011).

The core SCN also produces another type of neuropeptide that participates in maintaining cellular synchrony, behavioral rhythmicity, and entrainment—namely, GRP. In mice, GRP is expressed rhythmically under a light-dark cycle and act through the G-protein coupled receptor bombesin receptor 2 (BB2, also known as GRPR) (McArthur et al., 2000; Karatsoreos et al., 2006). Similar to VIP, application of exogenous GRP to the SCN produces phase shifts in a phase-dependent manner both *in vitro* and *in vivo* (Piggins et al., 1995; McArthur et al., 2000; Aida et al., 2002). This light-like response to GRP stimulation is accompanied by the upregulation of *Per1* and c-Fos expression in the dorsal SCN (Aida et al., 2002). GRP can also act as a secondary synchronizing neuropeptide when VIP-VPAC2 signaling is defective, since addition of GRP to *Vpac2^–/–^* SCN explants can transiently restore network synchrony (Brown et al., 2005; Maywood et al., 2011). Recent single cell RNAseq studies have found that GRP-expressing neurons are a subpopulation of VIP neurons: approximately one-quarter of VIP^+^ cells in the SCN co-express GRP (Todd et al., 2020; Wen et al., 2020).

Neuropeptides expressed by the shell SCN have also been shown to modulate circadian timekeeping. The most prevalent neuropeptide expressed by the shell is AVP. *Avp* is rhythmically expressed in mice, as its promoter contains E-box motifs for CLOCK:BMAL1 transactivation (Jin et al., 1999). When the AVP receptors *V1a* and *V1b* are genetically ablated, mutant mice become resistant to jetlag, re-entraining abruptly to shifts in the light-dark cycles (Yamaguchi et al., 2013). Examination of clock gene expression coupled with bioluminescence imaging of SCN explants revealed that *V1a^–/–^V1b^–/–^* SCN neurons show severely permutated phase order, and loss of intercellular synchrony following phase resetting (Yamaguchi et al., 2013). These results suggest that AVP-mediated interneuronal communication provides buffering toward abrupt external perturbations, and disruption of AVP-V1a/b signaling leads to a weakened oscillator. A mouse model with *Bmal1* deletion specifically in AVPergic neurons shows enhanced re-entrainment and lengthened behavioral period, possibly due to the combined attenuation of *Avp*, *Prok2*, and *Rgs16* expression in the SCN shell of these conditional knockout mice (Mieda et al., 2015). Deletion of CK1δ in AVP neurons also results in lengthened behavioral period and altered spatiotemporal pattern of PER2:LUC oscillations in SCN slices, indicating that AVP neurons can regulate SCN pacemaking (Mieda et al., 2016).

The majority of VIP^+^ and AVP^+^ neurons co-express the neuropeptide neuromedin S (NMS) (Todd et al., 2020; Wen et al., 2020). Although mice lacking NMS retain normal circadian rhythms *in vivo*, blocking vesicular transmission from NMS^+^ neurons disrupted the network synchrony of the SCN (Lee et al., 2015). Manipulating the expression of core clock genes, such as *Bmal1* and *Per2*, within NMS-expressing neurons is sufficient to disrupt molecular oscillations and behavioral rhythms (Lee et al., 2015). Conversely, overexpression of the *Clock*Δ*19* transgene in NMS-expressing neurons can lengthen circadian period *in vivo*, indicating that periodicity is dictated by this subset of SCN neurons (Lee et al., 2015). Similar pace-setting effects have been reported for DRD1a^+^ cells in the SCN, which represent ∼60% of all SCN cells (Smyllie et al., 2016). When floxed *Ck1*ε*^*Tau*^* alleles are excised from DRD1a^+^ neurons, ∼60% of the *Drd1a^*cre/*+^:Ck1*ε*^*Tau/Tau*^* temporally chimeric mice exhibited a free-running period that resembles wildtype animals (*Ck1*ε*^*WT/WT*^*), whereas ∼30% of the chimeric mice displayed a short period similar to *Ck1*ε*^*Tau/Tau*^* animals (Smyllie et al., 2016). The majority of chimeric SCN slices also displayed significantly lengthened PER2:LUC period, suggesting that DRD1a^+^ cells play a dominant role in period determination within the SCN (Smyllie et al., 2016).

## Embryonic Development of the Hypothalamus

Hypothalamic histogenesis follows the same general pattern as that observed in other neural tube-derived brain regions, with dividing progenitors residing in the ventricular zone and producing neuronal and glial precursors that migrate laterally into the parenchyma (Bedont and Blackshaw, 2015; Xie and Dorsky, 2017). It was originally thought that hypothalamic development follows an outside−in pattern, where lateral hypothalamic nuclei are generated first and displaced outward by medial nuclei that are born later (Shimada and Nakamura, 1973; Altman and Bayer, 1978). However, more recent research has found that in some hypothalamic regions, including the arcuate nucleus and dorsolateral anterior hypothalamus, cells occupying different medial-lateral locations are born during the same interval (Markakis and Swanson, 1997; Padilla et al., 2010).

After neural plate formation following gastrulation, diffusible morphogens generated by the mesodermal domains such as the notochord and prechordal plate (PCP) begin patterning the developing nervous system, including the presumptive hypothalamus. These morphogenic cues modulate important processes during hypothalamic induction and the establishment of regional identity (Bedont and Blackshaw, 2015). Sonic hedgehog (SHH) is a lipid−linked polypeptide signal that is first released from the prechordal plate, is necessary for induction of the hypothalamus, and drives *Shh* expression in the ventral diencephalon (Shimamura and Rubenstein, 1997). Through a Glioma associated oncogene(GLI)-mediated signaling cascade, PCP-derived SHH activates markers of hypothalamic identity along the overlying ventral diencephalic midline (Dale et al., 1997; Motoyama et al., 2003; Manning et al., 2006). Studies have shown that SOX2 and SOX3 can activate and maintain *Shh* transcription in the hypothalamic neuroepithelium through direct binding to the long-range Shh forebrain enhancer-2 (SBE2), whereas T-box transcription factors 2 (TBX2) and TBX3 repress *Shh* in the caudal hypothalamus by sequestering SOX2 away from the SBE2 (Zhao et al., 2012; Trowe et al., 2013). Sineoculis homeobox homolog 3 (SIX3) likewise targets the SBE2 to directly activate *Shh* transcription (Geng et al., 2008; Jeong et al., 2008). Using the *Nkx2.1-cre* mouse line, selective deletion of *Shh* from the basal plate domain of the developing hypothalamus results in ablation of markers of tuberal and anterior hypothalamic nuclei, along with thinning of the telencephalic and hypothalamic neuroepithelium (Shimogori et al., 2010). *Shh* deletion in the zona limitans interthalamica (ZLI) leads to a complete loss of prethalamic markers such as LIM homeobox 1 (*Lhx1)* and Gastrulation brain homeobox 2 (*Gbx2)* (Szabó et al., 2009a, b). *Shh* expression is also dependent on Retina and anterior neural fold homeobox (*Rax)*, as *Rax^–/–^* mouse embryos show a downregulation of *Shh* expression in the dorsomedial portion of the hypothalamus along with underdevelopment of the hypothalamic neuroepithelium (Orquera et al., 2016).

In addition to SHH, modulators of Wingless/Int-1 (WNT) signaling also regulate the patterning of the diencephalon and hypothalamus (Bedont and Blackshaw, 2015). The WNTs are a diverse family of secreted, palmitoleoylated signaling glycoproteins well known for their role in regulating anteroposterior patterning (Mikels and Nusse, 2006). WNTs released by the posterior neurectoderm and somites promote hindbrain fate, whereas the PCP and anterior neurectoderm produce WNT inhibitors to antagonize WNT signaling. *Wnt8b* is expressed in the mouse posterior hypothalamus beginning at ∼ E8.5, consistent with a role in patterning (Braun et al., 2003; Shimogori et al., 2010; Martinez-Ferre et al., 2013). *Wnt7a* and *Wnt7b* are expressed selectively in prethalamic and hypothalamic GABAergic neuronal progenitors around E12.5, suggesting a role in interneuron development; however, their function has not been well characterized (Shimogori et al., 2010). When the transcriptional repressor of WNT targets, Transcription factor 7-like 1 (*Tcf7l1)*, is conditionally knocked out in the mouse hypothalamus and pituitary, the developing hypothalamus is posteriorized (Gaston-Massuet et al., 2016). In contrast, loss-of-function of β-catenin (encoded by *Ctnnb1*) results in the anteriorization of the hypothalamus as seen in *Foxd1-Cre; Ctnnb1^*lox/lox*^* and *Nkx2.1-Cre; Ctnnb1^*lox/lox*^* mice (Newman et al., 2018b). Loss of SIX3, a direct *Wnt1* repressor, results in rostral expansion of caudal diencephalic markers as well as prosencephalon truncation (Lagutin et al., 2003). Lastly, *Lhx2*, a potential upstream inhibitor of WNT signaling (Peukert et al., 2011), has also been shown to play a role in the patterning of the telencephalic-optic-hypothalamic field and to specify SCN neurons at the expense of neuroendocrine fates (Roy et al., 2013).

## Embryonic Development of the SCN

**FIGURE 3:**
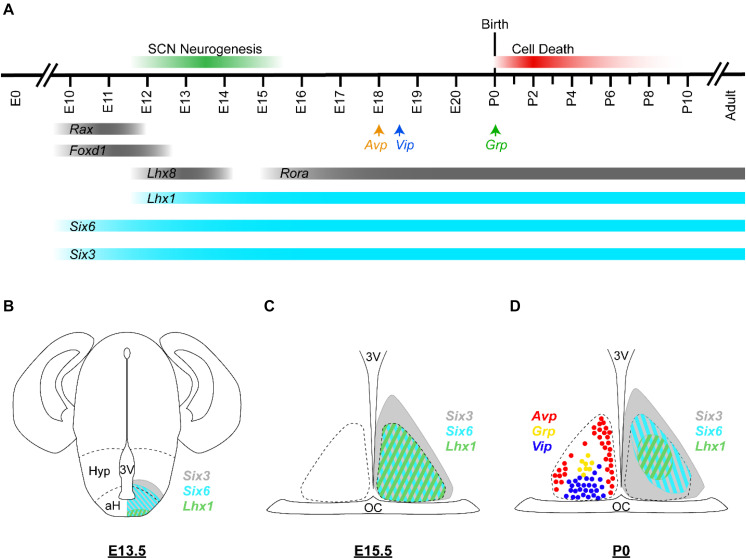
Embryonic development of the murine SCN. **(A)** Developmental timeline of the murine SCN. In mice, SCN neurogenesis begins around E12 and is considered complete by E15 (green bar). *Avp*, *Vip*, and *Grp* transcripts are first detected in the developing SCN at E18, E18.5, and P0, respectively (arrows). Many SCN cells are lost during P0–P8 by apoptotic cell death (red bar). Expression timeline of selected hypothalamus- and SCN-enriched transcription factors are shown; those with known developmental functions are depicted in turquoise and the others are depicted in gray. **(B–D)** Schematic illustrations of the murine brain/SCN showing the spatiotemporal expression patterns of *Six3* (gray), *Six6* (turquoise), and *Lhx1* (green) at **(B)** E13.5, **(C)** E15.5, and **(D)** P0. Cells expressing *Avp* (red), *Grp* (yellow), and *Vip* (blue) at P0 are depicted as colored circles. Dashed lines in **(A)** indicate the boundaries of the anterior hypothalamus (aH) and the hypothalamus (Hyp). Dashed lines in **(C,D)** indicate the margins of the SCN. Expression data are from Shimogori et al. (2010), VanDunk et al. (2011), Bedont et al. (2014), Newman et al. (2018a); unpublished observation (AH Cheng and HYM Cheng). 3V, third ventricle; OC, optic chiasm.

SCN neurogenesis begins at ∼60% of gestation in rodents (E12 for mice) (Figure 3A; Shimada and Nakamura, 1973; Antle et al., 2005; Kabrita and Davis, 2008). By ∼70% of gestation (E13.5), most ventrolateral neurons have already been produced, while dorsomedial neurogenesis is still underway. The final major burst of SCN neurogenesis (including a number of ventrolateral neurons) occurs at ∼80% of gestation (E15). Neurogenesis in the SCN along its rostral/caudal axis also shows heterochronicity, with neurogenesis in the medial SCN peaking at E12, in the caudal SCN peaking at E13.5, and in the rostral SCN peaking at E14. After immature SCN neurons are generated, they will continue to develop and ultimately express signature neuropeptides such as VIP, GRP, and AVP. Consistent with the regional differences in the peak timing of neurogenesis, cell types that are concentrated in the core SCN (e.g., VIP, GRP, and calbindin expressing cells) are mostly born early. In comparison, AVP neurons of the shell SCN are generated consistently during the period of neurogenesis that extends into later embryonic ages, with those in the middle-posterior regions generated prior to those situated in the anterior pole (Antle et al., 2005). It has been suggested that SCN core and shell neurons derive from distinct progenitor pools in the neuroepithelium (Altman and Bayer, 1986), but the precise mechanisms that regulate cell−type differences in the timing of SCN neurogenesis are not well understood.

SCN development is modulated by morphogen signaling. For instance, the WNT receptor frizzled 5 (*Fzd5*) is detected at E10.5–13.5 in mitotic cells (VanDunk et al., 2011). *Fzd5* is later downregulated, coinciding with the induction of distal-less homeobox 2 (*Dlx2*), which is a selector gene important for GABAergic neuron development (Pla et al., 2018). In addition, members of the fibroblast growth factor (*Fgf*) family are known to control the development of the hypothalamus and the SCN (Tsai et al., 2011). *Fgf8*, one of the 22 *Fgf* ligands, is expressed in the developing hypothalamus by E9.5 in developing mouse embryos, with robust expression in regions surrounding the optic chiasm (Fon Tacer et al., 2010). In homozygous *Fgf8* hypomorphic mice (∼54% reduction of *Fgf8* mRNA) at postnatal day (P)0, SCN volume as well as the expression of AVP and VIP are severely reduced, indicating that normal SCN development relies heavily on *Fgf8* signaling (Brooks et al., 2010; Tsai et al., 2011; Miller et al., 2016). Furthermore, hypomorphism in the cognate tyrosine kinase receptor of FGF8, *Fgfr1*, also causes reduction in VIP expression but does not affect SCN volume. The lesser dependence of SCN development on *Fgfr1* suggests possible compensation by other FGF receptors, as *Fgfr1*, *2*, and *3* are all expressed along the proliferative ventricular zone, but *Fgf8* is the only ligand expressed robustly in the ventral diencephalon near the presumptive SCN (Wanaka et al., 1990; Crossley and Martin, 1995; Belluardo et al., 1997; Bansal et al., 2003). Hence, the consequence of *Fgf8* expression deficit is more severe, as other compensatory factors may not be available during SCN development.

## Neuronal Differentiation In The Developing SCN

Many transcription factors such as *Rax*, forkhead box D1 (*Foxd1)*, *Nkx2.2*, *Lhx2*, *Six3*, *Six6*, and ventral anterior homeobox 1 (*Vax1*) are expressed in the ventral anterior hypothalamic neuroepithelium prior to the onset of SCN neurogenesis, although expression of many of these factors become restricted to specific anatomical regions or is lost entirely as the SCN develops (Shimogori et al., 2010; VanDunk et al., 2011).

A subset of these genetic markers such as *Lhx2*, *Rax*, *Foxd1*, and *Nkx2.2* are expressed transiently in the ventral anterior hypothalamus and are gradually lost as the SCN develops. *Foxd1* is broadly expressed in the developing hypothalamus from E11.5 to E13.5 and has been shown to be necessary for SCN development (Newman et al., 2018a). At the early stages of SCN neurogenesis, *Foxd1*-deficient mice display mild developmental deficits, with reduced expression of *Vax1* and *Six3* and a decrease in cellular proliferation (Newman et al., 2018a). By P0.5, more severe defects are observed in the SCN of *Foxd1*-deficient mice, including reduced expression of genetic markers and agenesis (Newman et al., 2018a). *Rax* is expressed in the murine ventral hypothalamus between E10.5 and E12.5 (Pak et al., 2014). Deletion of *Rax* prior to E8.5 disrupts *Shh* expression and is accompanied by a patterning defect of the mediobasal hypothalamus as described in the previous section, whereas later deletion causes misspecification of ventral medial hypothalamic nucleus (VMH) neurons (Lu et al., 2013; Orquera et al., 2016). *Lhx2* is required for specification of the SCN, as *Lhx2^–/–^* mice lack expression of multiple central anterior markers at E12.5, including *Lhx1 and Vax1* (Roy et al., 2013). In contrast to many early anterior hypothalamic markers that are down-regulated in the SCN later in development, *Six3* and *Six6* expression in the SCN persists throughout the lifespan (Shimogori et al., 2010; VanDunk et al., 2011; Clark et al., 2013). Both *Six3* and *Six6* are required for initial SCN specification, as both *Nestin-cre; Six3^*fl/fl*^* and *Six6^–/–^* mice fail to form a morphologically recognizable SCN or to express SCN-specific markers (VanDunk et al., 2011; Clark et al., 2013). Collectively, these observations suggest that early genetic markers have a long-lasting impact on SCN development by regulating the activation of other transcription factors. Moreover, they may be able to prime the activation of *cis*-regulatory elements that control the expression of cell type-specific genes during differentiation.

Downstream of *Six3* and *Six6*, *Lhx1* is a crucial regulator of SCN terminal differentiation (Figure 3; Bedont et al., 2014). *Lhx1* is considered to be the only known transcription factor involved in SCN differentiation and the earliest selective marker expressed throughout the developing SCN (VanDunk et al., 2011; Bedont et al., 2014). *Six3-cre; Lhx1^*fl/fl*^* SCN retains expression and proper compartmentalization of most markers initially expressed prior to E16.5, but neuropeptides with important roles in circadian function, including VIP, GRP, and AVP, are absent in the adult SCN (Bedont et al., 2014). By P4, significantly fewer SCN neurons are present in *Lhx1*-deficient SCN due to increased cell death from P0 to P4 (Bedont et al., 2014). Through bioinformatic and luciferase analyses, LHX1 has been shown to directly regulate the expression of *Vip* and potentially *Nms*, *Prok2*, and *proenkephalin* (Bedont et al., 2014; Hatori et al., 2014). Given that *Lhx1* is first detectable at E11.5, the relatively normal development of *Lhx1*-deficient SCN from E11.5 to E16.5 suggests that *Lhx1* is not necessary for early cell fate decisions or regional patterning of the SCN. Instead, *Lhx1* is likely regulating terminal differentiation of SCN neurons. It is also possible that the loss of *Lhx1* is compensated by one or more factors during this period; one potential candidate is *Lhx8*, an *Lhx1* homolog that is co-expressed with LHX1 in the SCN from E11.5 to E16.5 (Shimogori et al., 2010). Alternatively, *Creb3l1*, an *Lhx1*-regulated gene, is a regulator of AVP expression in the SCN, although its role in SCN development has yet to be explored (Greenwood et al., 2014; Hatori et al., 2014).

*Zfhx3* is another genetic marker with known roles in certain aspects of SCN function. *Zfhx3* is highly and almost exclusively expressed in the adult SCN (Lein et al., 2007). While the constitutive knockout of *Zfhx3* results in preweaning sublethality in heterozygous mice and the dominant missense mutation of *Zfhx3*, *Short circuit* (*Sci)*, is homozygous perinatal lethal, adult mice with a single allele of *Sci* show downregulated neuropeptide expression and shortened period of wheel-running activity (Parsons et al., 2015). Furthermore, ablating *Zfhx3* in adult mice using a tamoxifen-inducible transgenic line recapitulated the circadian behavioral deficit in *Zfhx3^*Sci/*+^* mice, indicating that *Zfhx3* acts to regulate SCN function in adulthood by activating transcription at AT motifs (Parsons et al., 2015; Wilcox et al., 2017). Its function in the developing SCN remains unclear.

The clock gene *Rora* is expressed in the ventral SCN starting at E14.5, throughout the SCN at E17.5, and in a pattern more restricted to the SCN shell by P21 (VanDunk et al., 2011; Newman et al., 2018a). Staggerer mice (*Rora*^*sg/sg*^) containing a deletion of the fifth exon of *Rora* have a morphologically normal SCN with nominal VIP and AVP expression; however, more characterization is necessary to conclude the function (or the lack thereof) of *Rora* during SCN development, as only the expression of VIP and AVP were examined in *Rora*^*sg/sg*^ mice (VanDunk et al., 2011).

## Neuronal Loss, Glial Development, and Synaptogenesis

A large number of SCN neurons are lost during the perinatal period through the activation of caspases and pro-apoptotic proteins of the Bcl-2 family. However, the mechanisms that initiate apoptosis in SCN neurons are largely unclear. SCN cell death begins as synapse formation increases, with substantial death between P1–P7 in mice (Ahern et al., 2013; Mosley et al., 2017). SCN neuronal survival might be dependent on intercellular communication, as apoptotic neurons are isolated from the neuronal clusters (Moore and Bernstein, 1989). Although peak cell death occurs in the murine SCN by P7, an additional 20% of cells are lost by adulthood (Bedont et al., 2014).

It has been estimated that the adult rat SCN contains at least 10^8^ synapses with the majority of these being intra-SCN connections (Güldner, 1976; van den Pol, 1980; Moore and Bernstein, 1989). Anatomical studies investigating synaptology in the rat or hamster SCN have revealed important insights on the timing of synapse formation within the SCN (Lenn et al., 1977; Moore and Bernstein, 1989; Laemle et al., 1991; Speh and Moore, 1993). Extensive studies in rats demonstrated that synaptic development is largely a postnatal event with a spike of synaptogenesis occurring between P4 and P10 (Moore and Bernstein, 1989). At E19, 2 days after the end of rat SCN neurogenesis, the neuropil surrounding SCN neurons is sparse and contains large and medium-sized dendritic profiles (Moore and Bernstein, 1989). Immature synapses with very few synaptic vesicles are also present at this time (Lenn et al., 1977; Moore and Bernstein, 1989). Synaptic number and diversity gradually increase from E21 to P2, and then expand rapidly from P2 to P10 (Moore and Bernstein, 1989). Another 30% of the synapses are formed after P10 (Moore and Bernstein, 1989). The window of SCN synaptogenesis overlaps with the arrival of prominent afferent projections. For both mice and rats, terminals from the RHT begin to sparsely innervate the ventrolateral SCN ipsilaterally at birth, followed by the first appearance of contralateral projections in the ventromedial SCN at P4 (McNeill et al., 2011). By P10, the morphology of the axon terminals and the density of the RHT projections are considered to be adult-like (McNeill et al., 2011). Other SCN afferents such as the raphe nuclei and the IGL also innervate the nuclei postnatally and slowly mature in the following weeks (Takatsuji et al., 1995; Migliarini et al., 2013).

Similar to other brain regions, astrogliogenesis follows neurogenesis in the SCN, with the astrocyte marker glial fibrillary acidic protein (GFAP) first detectable shortly before birth, at E15 in hamsters and E20 in rats (Botchkina and Morin, 1995; Munekawa et al., 2000). In both species, there is a postnatal increase in GFAP^+^ processes that complements the decreasing expression of vimentin (a marker of radial glia) (Botchkina and Morin, 1995; Munekawa et al., 2000). The first major increase in GFAP expression within the SCN, indicative of astrocytic maturation, occurs around P3-P4 in rats (Munekawa et al., 2000).

## Expression of Clock Genes and Neuropeptides

During late gestation, circadian rhythms of clock gene expression gradually emerge in a staggered fashion. PER proteins are generally considered to be the earliest core clock components to exhibit rhythmicity. PER2:LUC rhythms are detected as early as E13.5 in SCN slice cultures, but histological data collected from *in vivo* studies have placed the onset of *Per2* and PER2 rhythm at a later stage, around E17 (Shimomura et al., 2001; Ansari et al., 2009; Wreschnig et al., 2014; Landgraf et al., 2015; Carmona-Alcocer et al., 2018). Per1 gene and protein expression begin to show daily oscillations at around the same developmental age as *Per2*, or ∼E18 (Shimomura et al., 2001; Ansari et al., 2009). In comparison, *Cry1* and *Cry2* show robust rhythmicity after birth, with *Cry1*, CRY1, and CRY2 beginning to oscillate at P1, P10, and P2, respectively (Ansari et al., 2009; Huang et al., 2010). In contrast to the highly dynamic expression of PER and CRY, CLOCK and BMAL1 are constitutively expressed in the adult or developing murine SCN, respectively (Von Gall et al., 2003; Ansari et al., 2009). CLOCK is detectable at low levels at E18 and gradually rises to reach adult-level expression by P10 (Ansari et al., 2009). On the other hand, BMAL1 is robustly expressed in a large proportion of SCN cells at E18 and remains at a constantly high level during later developmental stages (Ansari et al., 2009). However, rhythmic *Bmal1* expression in the SCN can be detected at ∼P3 (Huang et al., 2010). The transcriptional activator of *Bmal1*, *Rora*, is first expressed at E13.5 and is found throughout the murine SCN by E17.5; therefore, *Rora* might contribute to the expression of BMAL1 at E18 (Ansari et al., 2009; VanDunk et al., 2011).

In mice, *Vip* expression is first detected ∼3.5 days after the end of neurogenesis in the SCN core, at E18.5 (Kabrita and Davis, 2008; VanDunk et al., 2011). VPAC2 and VIP reach detectable levels shortly after birth (P0 to P2) (Carmona-Alcocer et al., 2018). Further increases in the level of VIP expression as well as VIP-containing projections have been reported as the SCN matures (Herzog et al., 2000). Similarly, *Avp* is first detected in the murine SCN at E18 and robust expression of AVP is evident by P0 (Hyodo et al., 1992; VanDunk et al., 2011; Bedont et al., 2014). AVP expression continues to rise as the animal matures to ∼P30, at which point an adult level of expression is achieved (Herzog et al., 2000).

Intriguingly, mature SCN neurons continue to express genes that are commonly associated with maintenance of the stem cell states, even though the SCN does not undergo adult neurogenesis. These genes include doublecortin-like (DCL), transportin 1, *Six3*, *Lhx1*, and *Sox2* (Sato et al., 2011; VanDunk et al., 2011; Saaltink et al., 2012; Hoefflin and Carter, 2014; Brown et al., 2017; Beligala et al., 2018; Cheng et al., 2019). SCN neurons also conspicuously exhibit low levels of NeuN, a marker for mature neurons (Morin et al., 2011). This raises the possibility that SCN neurons are not fully committed to a differentiated state, thus retaining a degree of plasticity that presumably allows it to rearrange neuronal circuitry in order to entrain and adapt to a dynamic environment. It is also possible that SCN neurons have “re-purposed” these stem cell or developmental genes for circadian rhythm regulation once their canonical role has been fulfilled.

## Author Contributions

AHC wrote the manuscript. H-YMC edited the manuscript. Both authors contributed to the article and approved the submitted version.

## Conflict of Interest

The authors declare that the research was conducted in the absence of any commercial or financial relationships that could be construed as a potential conflict of interest.
